# Comparison of cd64 levels performed by the facs and accellix systems

**DOI:** 10.1186/2197-425X-3-S1-A1012

**Published:** 2015-10-01

**Authors:** CL Sprung, RC Morales, H Kasdan, A Reiter, S Keren, J Meissonnier

**Affiliations:** Hadassah Hebrew University Medical Center, Department of Anesthesiology and Critical Care Medicine, Jerusalem, Israel; LeukoDx, Jerusalem, Israel

## Background/Purpose

Medical flow cytometry (FACS) provides diagnostic answers by detecting the presence and concentration of cell populations, and/or by measuring concentrations of cell surface markers expressed on cells. Currently, FACS is limited to high complexity laboratories with time consuming pre-analytical steps, requiring trained technologists available only during business hours. The Accellix table top flow cytometer automates the process with results available within 20 minutes. Sample preparation and reading are performed in a dedicated disposable cartridge. Analytical data processing utilizing proprietary algorithms provides answers directly to the user.

## Methods

The Accellix disposable cartridge-based platform implements sample preparations using three reagent blisters. The three Accellix CD64 cartridge blisters contain staining cocktail of conjugated monoclonal antibodies, lysis buffer and reference beads respectively. Once sample processing is complete, the sample flows through a dedicated reading channel where data is acquired. The present study compared the results of neutrophil CD64 levels performed by standard laboratory FACS and the Accellix system in ICU infected and control patients and normal volunteers.

## Results

**Figure 1:**
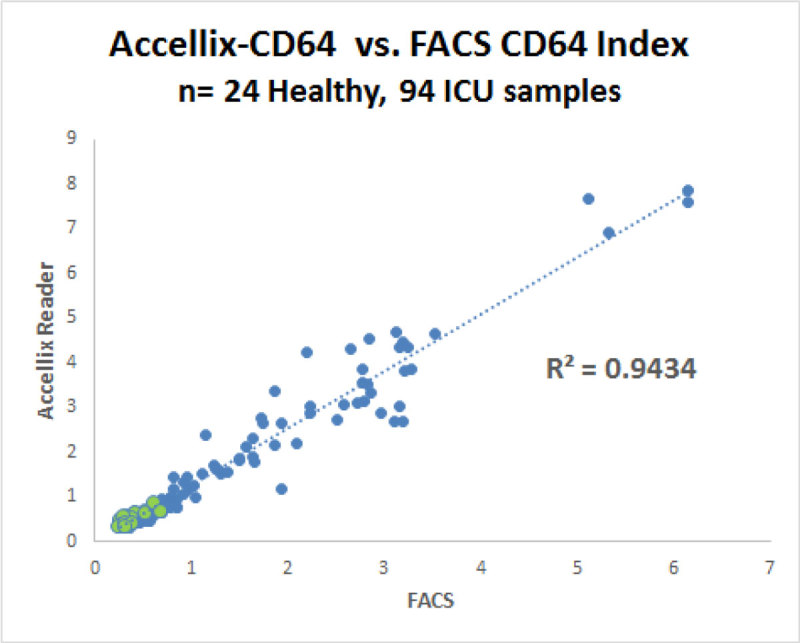
Figure 1

In a demonstration of cell surface marker quantitation a comparison study of 118 blood samples showed a correlation coefficient of 0.94 for Accellix determined neutrophil CD64 compared to those determined using a FACS. The comparison of the CD64 levels performed by the FACS and the Accellix system are shown in figure 1.

